# 6-Sulfo LacNAc (Slan) as a Marker for Non-classical Monocytes

**DOI:** 10.3389/fimmu.2019.02052

**Published:** 2019-09-13

**Authors:** Thomas P. Hofer, Arjan A. van de Loosdrecht, Christiane Stahl-Hennig, Marco A. Cassatella, Loems Ziegler-Heitbrock

**Affiliations:** ^1^Immunoanalytics Core Facility and RG Tissue Control of Immunocytes, Helmholtz Centre Munich, Munich, Germany; ^2^Department of Hematology, VU University Medical Center, Amsterdam, Netherlands; ^3^Deutsches Primatenzentrum, Göttingen, Germany; ^4^Section of General Pathology, Department of Medicine, University of Verona, Verona, Italy; ^5^Independent Researcher, Munich, Germany

**Keywords:** monocyte subsets, slan, man, monkey, inflammation, cancer, CMML, lymphoma

## Abstract

Monocytes are subdivided into three subsets, which have different phenotypic and functional characteristics and different roles in inflammation and malignancy. When in man CD14 and CD16 monoclonal antibodies are used to define these subsets, then the distinction of non-classical CD14low and intermediate CD14high monocytes requires setting a gate in what is a gradually changing level of CD14 expression. In the search for an additional marker to better dissect the two subsets we have explored the marker 6-sulfo LacNAc (slan). Slan is a carbohydrate residue originally described to be expressed on the cell surface of a type of dendritic cell in human blood. We elaborate herein that the features of slan+ cells are congruent with the features of CD16+ non-classical monocytes and that slan is a candidate marker for definition of non-classical monocytes. The use of this marker may help in studying the role of non-classical monocytes in health and in diagnosis and monitoring of disease.

## Introduction

The identification of monocytes in human blood has become much easier with advent of flow cytometry and the use of monoclonal antibodies to cell surface molecules. Antibodies to CD14 have been widely used for monocyte identification and with additional staining for CD16 at least three subsets (classical, intermediate, non-classical) can be defined (1). The CD14++CD16- classical monocytes can be clearly separated from the CD14++ CD16+ intermediate monocytes based on an isotype control for CD16 (2). However, the dissection of intermediate and non-classical monocytes is difficult and different approaches based on the level of CD14 expression have been used to set a cut-off between the two (2). Since differential roles in disease of these two CD16+ monocyte subsets have been documented, an unequivocal strategy is required for their dissection and here the use of the slan-marker has been suggested (3).

The slan-marker was first targeted with a monoclonal antibody termed M-DC8. This antibody was generated by immunizing Balb/c mice with human blood mononuclear cells depleted of T and B cells and monocytes (4). The resultant IgM antibody selectively stained about 1% of the mononuclear cells with light scatter properties between lymphocytes and monocytes. Phenotypic analysis of the M-DC8+ cells revealed that they had low CD33 and high CD16 expression levels.

Later on, the molecule recognized by the antibody was shown to be 6-sulfo LacNAc (slan), a sugar structure, which is linked to the cell surface protein PSGL-1 (P-selectin glycoprotein ligand), and the cells were dubbed slan dendritic cells (slanDCs) (5). There was early evidence suggesting that the blood leukocytes, which express the M-DC8 marker, belong to the monocyte lineage based on its similarity to the CD16+ monocytes including the low level expression of CD14 and absence of CCR2 (6, 7).

**Figure 1:**
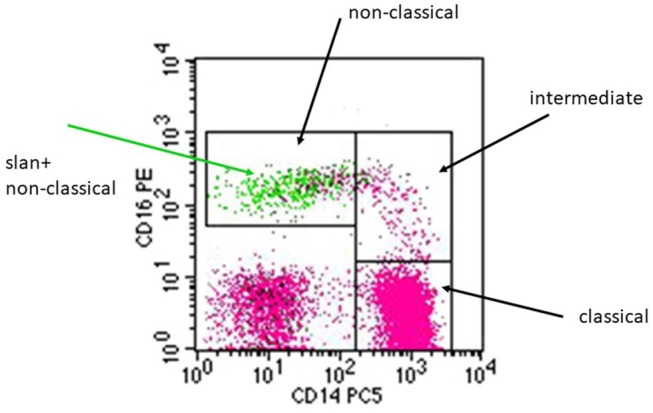
Illustration of the pattern of slan+ positive cells in a CD14 CD16 dot plot. A whole blood sample from a healthy donor was stained for CD14, CD16, DR, and for slan using the FITC-conjugated slan IgM antibody (# 130-117-371, Miltenyi Biotec). Black arrows indicate the monocyte subsets defined via CD14 CD16 staining. The green arrow points at the green dots that represent the slan+ monocytes, which localize within the non-classical monocyte gate.

As illustrated in a CD14 CD16 dot plot, the slan+ cells (green) localize to the gate of non-classical monocytes (Figure 1) and here they account for the majority of CD14+CD16++ monocytes. There are a few events within that gate, which are slan-negative (pink color).

In phenotypic analyses similar patterns of cell surface markers were noted for CD16+ monocytes and “slanDCs.” Also, similar results were reported for functional analyses such as cytokine production and antigen presentation. The same applies to many clinical studies and to response to anti-inflammatory therapies.

With the advent of transcriptome analysis, unsupervised hierarchical clustering approaches have then demonstrated that the blood slan+ cells cluster with monocytes and not with dendritic cells (8, 9). These findings have provided additional strong evidence for the monocyte nature of the slan+ leukocytes.

In the following, we will summarize the arguments to show that in human blood, slan+ cells are a subset of the CD16+ monocytes. Further, we will argue that slan is an appropriate marker for non-classical monocytes.

## Cell Surface Phenotype of CD16+ Monocytes and slan+ Cells

As mentioned, the slan-residue is a sugar structure attached to PSGL1. PSGL1 is expressed by all leukocytes (10) including CD16- and CD16+ monocytes (11) but the slan residue is only found on a subset of CD16+ monocytes. It was shown that CHST2 can link the residue to the PSGL1 protein molecule (5). There are additional transferases including CHST15, which similar to CHST2 shows increased mRNA expression in CD16+ monocytes (12) and B3GALT2, which is increased in slan+ compared to slan- CD16+ monocytes (13). These findings need confirmation and the role of these transferases in generating the slan-residue needs to be determined.

CD16+ monocytes have been characterized for cell surface markers in a host of flow cytometry analyses. Compared to CD14++ monocytes, higher levels of expression for HLA-DR and lower levels for CD11b, CD14, CD33, and CD64 have been noted for these cells (14, 15). Also, CD11a, c, and d were higher on the CD16+ cells, while CD62L was essentially absent. With respect to chemokine receptors, the CD16+monocytes were found to be CCR2 negative (11, 16) while CX3CR1 was found increased (17) and this went along with an absent and an increased response to the respective chemokine. Finally, CD115 the receptor for macrophage colony stimulating factor (M-CSF-R) was found increased in CD16+ monocytes (18).

Looking at slan+ cells in blood, high levels of HLA-DR, CD11c, CD16, and CX3CR1 and low levels of CD11b, CD14, CD33, and CD64 were noted, while CD62L and CCR2 were absent (4, 7, 19). High levels of CD115 on blood slan+ cells were only reported recently (20, 21). Slan+ cells were shown to express receptors for C3a and C5a (5, 9, 22), while for CD16+ monocytes only expression of C3aR mRNA was noted [see table S5 in (23)].

Finally, C-type lectin receptors CD368 (Dectin-3) and CLEC5A were found essentially absent both in CD16+ monocytes and in slan+ cells, while classical monocytes showed a strong expression of these markers (24). Looking at these data, it is evident that the pattern of cell surface markers for the CD16+ monocytes and the slan+ cells is very similar. The congruent expression of these various functionally relevant receptors suggests similar functional properties of these cells.

## Function of CD16+ Monocytes and slan+ Cells

### Cytokine Production

In response to LPS (lipopolysaccharide) the CD16+ monocytes were shown to be potent producers of cytokines like TNF (tumor necrosis factor) (25), while the production of the anti-inflammatory cytokine IL-10 was decreased compared to classical monocytes (26). This pattern of high TNF and low IL-10 production in response to LPS was confirmed by others (18, 27, 28). Also, higher TNF production by CD16+ monocytes was seen after stimulation with TLR7/8 ligands, with *Aspergillus fumigatus* conidia and toxoplasma tachyzoites (27, 29, 30). Also for blood slan+ cells, high levels of IL-1, IL-6, IL-12, and TNF protein were reported after stimulation by toll-like receptor ligands (31–34). In addition, TNF levels were shown to be even higher in slan+ cells of HIV-infected individuals (35).

With respect to IL-10, slan+ cells were shown to express lower levels compared to slan- cells (31) and also compared to classical monocytes (21). This latter study, in fact, provided a side-by-side comparison of slan+ cells and CD16+ non-classical monocytes with respect to cytokine production and it confirmed the higher levels of TNF and IL-12 and the lower levels for IL-10 for both CD16+ non-classical monocytes and slan+ cells as compared to classical monocytes. Hence, the two cells share a characteristic cytokine production pattern with high TNF and IL-12 and low IL-10 expression and this includes a stronger responsiveness to the IFN-gamma-mediated priming compared to classical monocytes (21).

Since TNF and IL-12 play a dominant role in most inflammatory diseases, the concepts regarding the pathophysiological role of slan+ non-classical monocytes revolve around their ability to produce these cytokines. Because of this ability, the slan+ cells may be major players in infection and inflammation. Experiments, which selectively target these cells in disease models, are required to support this concept.

#### Cell-Cell Interactions

CD16+ monocytes in their original description were noted to express high levels of HLA-DR, i.e., the major MHC class II molecule in man (14). Consistent with the role of HLA-DR in presentation of peptide antigens to T cells, the CD16+ cells show potent induction of IFN-gamma in T cells in response to influenza Type A-antigen and purified protein derivative (36).

For the slan+ cells, antigen presentation studies using keyhole limpet hemocyanin and tetanus toxoid showed efficient induction of T proliferation (5). Here, the response generated by slan+ presenting cells was comparable to the response induced by CD11c+ dendritic cells and this was taken to support the conclusion that the slan+ cells belong to the dendritic cell lineage.

The induction of TH17 cells was shown to be supported both by CD16-positive monocytes and by slan+ cells. When CD4+ T cells were incubated in the presence of LPS with monocyte subsets then CD16+ intermediate monocytes were most potently supporting the generation of IL-17-producing T cells (28). In another study, using superantigen for T cell activation, the CD16+ non-classical monocytes were the strongest inducer of TH17 cells (37). Looking at slan+ cells, these cells were shown to be more potent than CD1c+ dendritic cells in inducing IL-17 in CD4+ CD45RA+ T cells after 7 days of co-culture (19).

In antibody dependent cellular cytotoxicity (ADCC), an effector cell can kill another cell via a bridging antibody that binds to the Fc-receptor on the effector cell and the cell surface antigen of a target cell. Monocytes are equipped with both high and low affinity Fc-receptors for IgG and the CD16+ blood monocytes were shown to efficiently kill B cell lymphoma cells via a CD20 monoclonal antibody (38). CD20-mediated ADCC of lymphoma cells was demonstrated for slan+ cells taken from healthy donors or patients with diffuse large B-cell lymphoma (39).

Furthermore, CD16+ monocytes showed ADCC against cells of the SKBR3 breast cancer cell line mediated via a monoclonal against HER2 (human epidermal growth factor receptor 2) (38). Strong ADCC activity against the same breast cancer cell line with the same anti-HER2 monoclonal antibody had been reported earlier when studying slan+ cells (40).

In the context of malignant melanoma, CD16+ non-classical monocytes were shown to be crucial to immune check-point blockade in that they mediated the killing of regulatory T cells via an antibody against CTLA-4 (cytotoxic T lymphocyte–associated antigen 4) (41). In this study, only patients with high numbers of CD16+ non-classical monocytes showed a decrease in tumor burden in response to therapy. This type of activity has not been reported from the perspective of slan+ cells, as yet.

Both CD16+ non-classical monocytes and slan+ cells have been noted to express the CD16 and CD32 Fc-receptors for IgG but none or little of the high affinity CD64 IgG Fc-receptor. In the context of ADCC, cooperation of CD16 and CD32 has been noted, but there was no role for CD64 (38). For slan+ cells such a cooperation of CD16 and CD32 had been reported earlier (40).

Slan+ cells have been shown to interact with neutrophils leading to an increased production of IL-12 by slan+ cells incubated with LPS plus IFNg (42). Also, neutrophils will reduce the cell death of slan+ cells, which occurs in *in-vitro* co-culture in the presence of LPS (43). Both induction of IL-12 and protection from cells death requires cell-cell contact. In this context, the CD16+ monocytes also have been noted to be susceptible to cell death in culture (44), but a protective effect of neutrophils or an induction of IL-12 has not been reported for CD16+ non-classical monocytes.

Conversely, slan+ cells can activate NK cells via IL-12 (42, 45). Such an activity has not been shown for CD16+ monocytes but it would not come unexpected since these cells are major producers of IL12 (46) and IL-12 is a major NK cell activator (47). Also, the activation of NK cells via transmembrane TNF expressed by slan+ cells (48) has not been shown for CD16+ monocytes yet but given the superior TNF production by CD16+ monocytes it is conceivable that these cells would be able to activate NK cells via this route.

One crucial issue in monocyte biology is the interaction of these cells with vascular endothelium. In *in-vitro* experiments human non-classical and also classical monocytes were reported to show a crawling (“patrolling”) behavior (49). No such data are available for slan+ cells. Transmigration across endothelium was shown for CD16+ monocytes and, interestingly slan+ monocytes were mentioned to do the same (50). While PSGL-1 is involved in leukocyte-endothelium-interaction (51), there is no report on the function of the slan residue on PSGL-1, albeit an interaction of slan with lectins and a role in monocyte-endothelial-interaction are likely.

Taken together the interactions with T cells reported for slan+ blood cells under the label “dendritic cells” have also been published for CD16+ monocytes. Also with respect to ADCC similar findings have been reported from the perspective of CD16+ monocytes and the slan+ cells. However, when it comes to interaction with NK cells and neutrophils then the “slan DC” studies provide novel insights for the CD16+ non-classical monocytes.

### Transcriptome of CD16+ Monocytes and Slan+ Cells

A comparative transcriptome study looked at CD1c+ dendritic cells, at classical, intermediate and non-classical monocytes and at slan-positive CD16+ and slan-negative CD16+ monocytes. Here, unsupervised hierarchical clustering clearly demonstrated that the slan+ cells cluster with monocytes and not the CD1c+ DCs (8).

Another study used hybridization to a human transcriptome array using cells isolated via magnetic cell sorting and flow cytometry cell sorting. Here the slan+ cells clustered away from both the CD1c+ and CD141+ DCs (9).

These transcriptome data consolidate the conclusion that slan+ cells in blood belong to the monocyte lineage. Therefore, at this stage the features previously described under the M-DC8+/slan+ dendritic cell concept, can now be ascribed to the slan+ non-classical monocytes. Therefore, in this paper the term “slan+ non-classical monocyte” will be used from here on.

### Flow Cytometry Approach to Slan+ Monocytes

**Figure 2:**
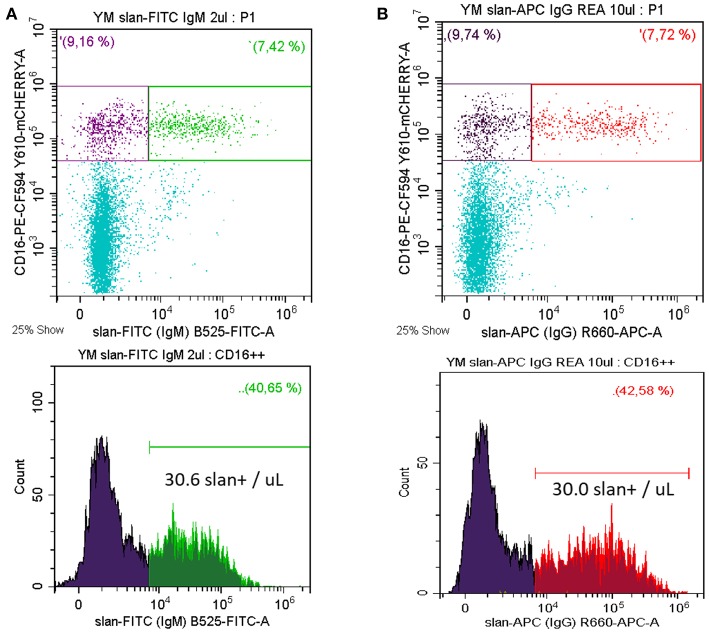
Whole blood staining for slan+ non-classical monocytes. Whole blood samples were stained for CD14, CD16, DR, and slan. Shown is the slan vs. CD16 staining of all CD14+ monocytes in the upper panels. The respective single parameter slan+ histogram for all CD16+ monocytes is given in the lower panel **(A)** FITC-conjugated slan IgM antibody (# 130-117-371, Miltenyi Biotec) **(B)** APC-conjugated slan human IgG antibody (# 130-117-919, Miltenyi Biotec, kindly provided by Miltenyi Biotec). In average of 5 donors the absolute number of FITC-mu-IgM slan+ cells was 37.6 ± 11.4 cells/μL and of APC-hu-IgG slan+ cells it was 39.6 ± 16.8 cells/μL. Venous blood samples were obtained from healthy volunteers after informed consent and with the approval of the Ethics Committee of the LMU Medical Faculty, Munich.

Monocytes currently are subdivided into three subsets, i.e., classical, intermediate, and non-classical monocytes (1) and in man they are defined using markers CD14, CD16, and DR. Separating non-classical and intermediate monocytes has been difficult within this setting, since different cut-off levels for CD14 have been used. To resolve this, the slan marker has been proposed as an additional marker for a positive definition of non-classical monocytes (18). In fact, molecular and clinical studies have demonstrated the feasibility of this approach (8). A typical staining of whole blood for slan+ non-classical monocytes is shown in Figure 2A. Here, we use a CD14 CD16 DR staining to determine the CD16 monocytes and then the slan+ CD16+ cells are defined. In the example shown there are 30.0 slan+ CD16+ cells /μL. In average of *n* = 5 the absolute number of these cells is 37.6 ± 11.4 cells/μL for the mouse IgM antibody.

Consistent with the carbohydrate nature of the slan structure the antibodies generated in the mouse are of the IgM class (4). More recently, a recombinant human IgG1 antibody has been generated at Miltenyi Biotec. This reagent, compared to isotype control, gives a similar staining pattern in flow cytometry (see Figure 2B).

Together, these data illustrate a straightforward strategy for determination of slan+ non-classical monocytes, a strategy that might be useful when it comes to monitoring of non-classical monocytes in disease and during therapy.

Recently it has been suggested that there might be subsets of slan+ non-classical monocytes with one subset characterized by an increase in expression of genes like CD41 and CD61 (13). Since these genes encode typical platelet receptors, the nature of this increase still needs to be resolved.

Also, we have to be open to the possibility that there may by some slan-negative cells with features of non-classical monocytes.

There have been reports that described CD16+ dendritic cells, which were identified among lineage-negative DR+ cells (23, 52, 53). While CD14-positive monocytes were excluded in the definition of these cells, the very low CD14-positive monocytes remained within the lineage-negative population. Comparative studies by Calzetti et al. have then demonstrated that cells dubbed CD16+ DCs do, in fact, belong to the CD16+ slan+ non-classical monocytes (21).

### Clinical Studies Involving Slan+ Non-classical Monocytes

While there is a host of studies on monocyte subsets in inflammation and cancer, we will herein only highlight selected studies relevant to slan+ cells. When it comes to increases and decreases of the number of slan+ cells in patients, then changes with gender and age in healthy donor control values need to be considered. Here, slan+ monocytes were shown to be significantly higher in infants aged 6–12 months and in the elderly at age 60–70y (54).

#### Chronic Myelomonocytic Leukemia

The definition of monocyte subsets has emerged as a diagnostic tool for chronic myelomonocytic leukemia (CMML). The WHO classification lists CMML among the myelodysplastic/myeloproliferative neoplasms and requires for diagnosis a persistent blood monocyte count >1,000/μL and > 10% of all blood leukocytes (55). Since monocytosis is not unique to CMML and since cases may present with subthreshold monocyte counts, novel diagnostic approaches were explored. The original finding by Vuckovic et al. (56) noted that “The CD14lowCD16+ monocyte subpopulation was not found in CMML patients.” Selimoglu-Buet et al. (57) then studied the diagnostic potential of this lack of non-classical monocytes by looking at the complementary increase of the classical monocytes and defining >94% of classical monocytes as a criterion for CMML. The usefulness of this additional test for diagnosis of CMML was subsequently confirmed (58). Furthermore, myelo-dysplastic syndrome (MDS) patients with subthreshold monocytosis, but increased classical monocytes were labeled “CMML-like” MDS and it was shown that several of these patients developed overt CMML within 1 year (59). Instead of looking at the increase of classical monocytes Hudson et al. (60) focused on the decrease of non-classical monocytes and reported a higher diagnostic specificity. Along these lines, Tarfi et al. (61) have then reported that slan-defined non-classical monocytes also gave a high diagnostic specificity. Hence, the slan marker may become the preferred tool in diagnosis of CMML based on the characteristic depletion of this subset.

Currently, a multicenter prospective ELN study is ongoing to validate the use of monocyte subsets in CMML diagnosis. To this end, the European Hematology Association and the European LeukemiaNet recommends the determination of monocyte subsets in flow cytometry to separate CMML from reactive monocytosis (62).

#### Cardiovascular Disease

Slan-defined monocyte subsets may be informative in atherosclerosis. Along these lines, Hamers et al. noted in a small study an increase of slan+ non-classical monocytes in patients with severe as compared to mild coronary artery disease (13). Also, an increase of slan+ cells had been noted in patients with peripheral artery disease (63). Given the many studies on the role of intermediate monocytes in cardiovascular disease, including their prognostic value (64), there also is potential for intermediate monocytes defined as CD16+ slan-negative monocytes in this context.

### Inflammatory Disease

In systemic lupus erythematosus (SLE) immune complexes are of central pathogenic importance and such complexes can recruit leukocytes and thereby initiate damage. For lupus nephritis with pronounced sub-epithelial immune complex deposits (class III and IV according to the International Society of Nephrology/Renal Pathology Society classification) an increased number of CD16+ cells had already been documented (65). Consistent with these findings in a recent study on lupus nephritis an increase in the frequency of slan+ monocytes in class III and IV glomeruli was shown (66). These slan data are obviously much more informative compared to staining for CD16+ cells because they strongly suggest the presence of non-classical monocytes while the demonstration of CD16+ cells in tissue sections is less specific since this receptor is also present on neutrophils and NK cells.

#### Cancer

An increased number of blood slan+ non-classical monocytes, associated with a decreased frequency of pDCs, has been found in patients with colorectal carcinoma (CRC) (22) and in diffuse large B cell lymphoma (DLBCL) (39).

In lymph nodes in proximity to metastatic carcinoma cells (where they are well-positioned for tumor cell destruction) slan+ cells can be readily detected (22). However, slan+ cells are not present within the primary sites nor within the metastases tissue in solid cancer.

In contrast to solid tumors, the slan+ cells can be found within lymphoma tissue and here they can display either dendrites that extend into the tissue or they have a more rounded macrophage-like morphology (39). The latter type of cell may be involved in antibody-dependent cellular phagocytosis (ADCP) of tumor cells. In addition, slan+ non-classical monocytes can efficiently destroy B lymphoma cells via anti-CD20 in ADCC (39).

Taken together there are several reports on slan+ non-classical monocytes in disease settings. Given the extensive literature on CD16+ monocytes in inflammation and cancer revisiting these areas with the use of the slan marker may generate novel insight into the monocyte subsets involved.

## Drugs Targeting slan+ Non-classical Monocytes

Glucocorticoid therapy was shown to selectively reduce the number of CD16+ monocytes, while classical monocytes increase and this was shown both in multiple sclerosis patients and in healthy volunteers (67, 68).

The depletion of CD16+ monocytes is likely to be mediated by induction of apoptosis and was shown to act via the nuclear steroid receptor (68). In a more recent article, the effect of high dose GCs on slan+ cells was studied in multiple sclerosis and here a depletion of these cells in blood was described (69).

Interferon-beta (IFN-beta) therapy in multiple sclerosis patients had been shown to decrease CD16 monocytes with low level expression of CD14 after 4 weeks of therapy (70). Later, such treatment was demonstrated to reduce blood slan+ cells in multiple sclerosis patients (69). These findings were substantiated in a study on hepatitis C patients, which showed an almost complete disappearance of slan+ cells and of CD14low CD16++ monocytes on day 30 of IFN-alpha therapy (71).

An anti M-CSF antibody in a rheumatoid arthritis pilot study showed depletion of both CD16+ non-classical and intermediate monocytes (72). Similarly, in Diffuse Type Tenosynovial Giant Cell Tumor (= Pigmented Villonodular Synovitis) a selective reduction of non-classical monocytes was noted after treatment with the humanized anti-M-CSF receptor antibody emactuzumab, a treatment that reduces the tumor–promoting macrophages within the tumor tissue (73). As detailed before, slan+ are rare among the tumor infiltrating M-CSF-R+ macrophages (74). Therefore, it is unlikely that these slan+ cells in tissue are an important therapeutic target in cancer. Still, the determination of slan+ non-classical monocytes in blood may be a useful tool for monitoring of anti-M-CSFR therapy in cancer.

G-CSF treatment can increase the number of slan+ monocytes (75, 76) and this is in line with earlier studies that suggested an increase of CD16 on total blood monocytes after G-CSF (77).

Lenalidomide is a thalidomide derivative used in therapy of multiple myeloma (78). It binds to the E3 ubiquitin ligase complex and directs its substrate specificity to IKFZ transcription factors leading to their proteasomal degradation (79) and this leads to cell death of myeloma cells. Lenalidomide treatment also leads to depletion of B cells and of non-classical monocytes, which correlates with the intracellular depletion of IKFZ1 protein (80). Earlier work suggest that ubiquitin is relevant to slan+ non-classical monocytes since transcriptome analysis of these cells has revealed a ubiquitin-signature in that altogether 50 UBC-linked genes were selectively up- or down-regulated in these cells (8). It remains to be determined whether any of these differential genes is involved in the lenalidomide depletion of slan+ non-classical monocytes

Laquinimod is a quinolone-3-carboxamide, which is being evaluated as a therapy for multiple sclerosis (81). In a phase I dose escalation study a reduction within 2 weeks of the slan+ cell frequency by 80% was noted (82), while in a separate study no change was seen for numbers of T cells, B cells, NK cells and CD14+ monocytes (83). The mechanism of action remains unclear, but an involvement of the aryl hydrocarbon receptor and of NF-kB has been proposed (82).

Taken together, most studies on drug effects have reported on monocyte subsets defined via CD14 and CD16 but only some have looked at slan+ cells. The slan-marker offers an unequivocal alternative for drug monitoring of non-classical monocytes in blood under various clinical settings.

### Slan+ Cells in Tissue

Another intriguing novel aspect is the detection of slan+ cells in various tissues. In normal tissue, these cells are sparse with a few scattered slan+ cells for instance in the dermis. However, in inflammatory disease such as psoriasis and atopic dermatitis these cells can increase substantially (19, 34, 84).

In tonsils, there is also a low number of these cells but at levels similar to CD141+ dendritic cells (20). Here, the cells localize preferably to the T cell areas (31). Phenotypically the tonsil slan+ cells show lower CD16 and CX3CR1 and higher CD14 compared to blood slan+ non-classical monocytes (20). Of note, the tonsils studied were from patients undergoing tonsillectomy for recurrent infection such that here information is only available on inflamed tissue. Therefore, it is unclear as to whether these differences are due to location or to inflammation or to both.

Also in lymph nodes, slan+ cells are rare but as discussed above they increase with metastasis of carcinomas to the draining lymph nodes (22).

The expression of the slan-marker in skin, tonsils, lymph nodes, and tumor metastasis as presented above would be consistent with the concept that these cells are the progeny of the blood slan+ non-classical monocytes, which have migrated into these tissues. On the other hand, it is possible that the slan-residue is induced via sulfo-transferases in an unrelated type of leukocyte residing in the tissue. However, experiments using tumor-cell conditioned media revealed that the slan marker is very stable and apparently not inducible in other leukocyte populations (22).

In any case, to resolve the relationship of slan+ monocytes in blood and slan+ cells in tissue, a comparative characterization including transcriptomics of slan+ cells in blood and tissue is required.

### Slan+ Cells in Other Species

**Figure 3:**
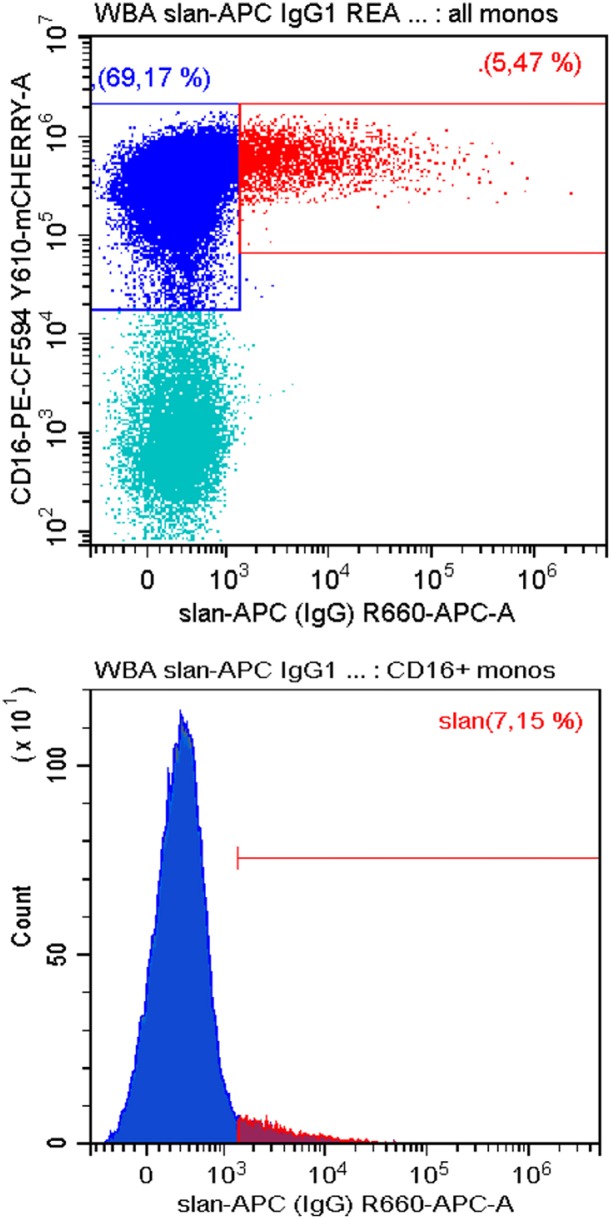
Staining for slan+ non-classical monocytes in common marmosets. Peripheral blood mononuclear cells from −140°C-stored samples of common marmosets (Callithrix jacchus) (Deutsches Primatenzentrum, Goettingen) were thawed and stained for CD14, CD16, CD56, DR, and slan (APC-conjugated slan human IgG antibody REA 1050, # 130-117-919, Miltenyi Biotec). Shown is the slan vs. CD16 staining of all CD14+ monocytes in the upper panel. The respective single parameter slan+ histogram for all CD16+ monocytes is given in the lower panels. CD56+NK cells were excluded. One of three samples is shown. In average of 3 samples the slan+ monocytes account for 3.4 ± 1.8% of all monocytes. Blood sampling was approved by the German Primate Center Ethics Committee and the Lower Saxony State Office for Consumer Protection and Food Safety in accordance with the European Union guidelines on the welfare of non-human primates used in Research and the European Union (EU directive 2010/63/EU).

To date all studies on slan+ monocytes have been conducted with human samples. We have tested whether the slan-antibodies can also be used to identify homologous cells in old-world and new world monkeys. Here, monoclonal antibodies targeting human cells surface molecules have been successfully used to define monocyte subsets based on CD14 CD16 markers (85). As shown in Figure 3 in blood mononuclear cells from common marmosets slan+ cells can be readily detected using the recombinant monoclonal antibody. Here a large proportion of monocytes is CD16 positive such that the percentage of slan+ cells among all CD16+ monocytes is low at 7.2%. However, the percentage of the slan+ cells among all monocytes is at 5.5% and with that comparable to man (see Figure 2B upper panel).

These data demonstrate that the new world marmoset monkeys have the potential to serve as a model in the study of slan+ monocytes.

It remains to be determined whether slan or a similar sugar structure exists on PSGL in other mammalian animals including mice.

## Concluding Remarks and Perspective

This review summarizes the evidence, which shows that the slan+ cells in human blood are part of the CD16+ monocytes and their phenotypic and functional properties are identical to non-classical monocytes. It remains to be determined whether slan covers all non-classical monocytes and whether there is heterogeneity among the slan+ cells. Single cell sequencing may be able to address these questions.

In any event, the slan-marker has potential for monitoring of non-classical monocytes in various disease states and the many studies on CD16+ monocytes in inflammation and cancer should be revisited using slan.

Future work should look into selective targeting of these cells in order to demonstrate a crucial role of slan+ non-classical monocytes and their cytokine production in disease. Then there are many open questions regarding the interaction of slan+ monocytes with the endothelium. Finally, it will be important to determine whether there is a structure homologous to slan on mouse non-classical monocytes such that these cells can be studied in experimental animals other than non-human primates.

## Author Contributions

TH and CS-H performed experiments. TH, AL, CS-H, MC, and LZ-H wrote the paper.

### Conflict of Interest Statement

LZ-H was provided with free slan reagents from Miltenyi Biotec for this project. The remaining authors declare that the research was conducted in the absence of any commercial or financial relationships that could be construed as a potential conflict of interest.
